# Angelman syndrome-associated ubiquitin ligase UBE3A/E6AP mutants interfere with the proteolytic activity of the proteasome

**DOI:** 10.1038/cddis.2014.572

**Published:** 2015-01-29

**Authors:** V Tomaić, L Banks

**Affiliations:** 1 Tumor Virology Research Group, International Centre for Genetic Engineering and Biotechnology, Padriciano 99, Trieste, Italy

## Abstract

Angelman syndrome, a severe neurodevelopmental disease, occurs primarily due to genetic defects, which cause lack of expression or mutations in the wild-type E6AP/UBE3A protein. A proportion of the Angelman syndrome patients bear UBE3A point mutations, which do not interfere with the expression of the full-length protein, however, these individuals still develop physiological conditions of the disease. Interestingly, most of these mutations are catalytically defective, thereby indicating the importance of UBE3A enzymatic activity role in the Angelman syndrome pathology. In this study, we show that Angelman syndrome-associated mutants interact strongly with the proteasome via the S5a proteasomal subunit, resulting in an overall inhibitory effect on the proteolytic activity of the proteasome. Our results suggest that mutated catalytically inactive forms of UBE3A may cause defects in overall proteasome function, which could have an important role in the Angelman syndrome pathology.

Ubiquitination is a highly specific process that involves a group of proteins responsible for adding ubiquitin molecules to cellular substrates, thereby resulting in the modulation of numerous cellular pathways.^1^ The deregulation of components of the ubiquitin conjugation system causes defects in many cellular functions and these have been associated with human pathogenesis.^2^ Of the components involved in the ubiquitin cascade, the E3 ubiquitin ligases provide the substrate specificity. By attaching ubiquitin molecules to their substrates, E3 ligases have direct control over the functions and, in many cases, protein turnover of these substrates. In addition, loss of function in a number of E3 enzymes has been shown to have an important role in the development of severe physiological conditions such as certain cancers and neurological disorders.^3^ A representative instance of the latter is Angelman syndrome (AS), a severe neurodevelopmental disorder, with clinical features of mental retardation, developmental delay, ataxia and epilepsy.^4, 5^ The principal protein affected in AS is the E3 ubiquitin ligase E6-associated protein (E6AP/UBE3A), the gene being found on chromosome 15q11-13. UBE3A was initially identified as an interacting partner of high-risk HPV-16 and -18 E6 oncoproteins,^6, 7^ but was subsequently found to be linked to the development of AS. AS develops mainly due to genetic defects that lead to the loss of expression of the maternal allele of the UBE3A gene in the hypothalamus.^8, 9^ Between 65 and 75% of AS patients have been diagnosed with the deletions of 15q11-13, 3–7% of patients show uniparental disomy and ~3% of cases have been found with imprinting defects, such that the functionally defective maternal copy of the gene is expressed in the brain.^5^ In addition, there are also 5–11% of individuals with AS whose sequence analyses show UBE3A mutations. Most of these have in-frame deletions that would be predicted to result in protein truncations,^10, 11^ but a number of those patients have milder mutations, such as point mutations, that do not affect the expression of the full-length protein.^12, 13^ The majority of these mutations however are defective in ubiquitin ligase activity, indicating that the loss of enzymatic activity of UBE3A is important in promoting the development of AS.^14^

Studies have demonstrated that ubiquitin ligase activity of UBE3A has a role in the proteasome-dependent degradation of several cellular substrates, and it can be reasoned that defects in the regulation of some of these substrates can contribute to AS development. However, although a number of UBE3A target proteins have been identified, including Sox9, C/EBP*α*, *α*-Synuclein, p27, promyelocytic leukemia (PML) tumor suppressor, annexin A1, amplified in breast cancer 1 (AIB1) and HHR23A,^15, 16, 17, 18, 19, 20, 21, 22^ characterization of their interactions with UBE3A have only partially contributed to an understanding of the molecular mechanisms behind the development of AS pathology. In addition, UBE3A has also been shown to interact with other components of the proteasome degradatory pathway, including the ubiquitin ligases HERC2, Ring1B and EDD,^23, 24, 25^ and recent studies demonstrated a direct interaction between UBE3A and the proteasome itself.^26, 27^ Whether any of these interactions might also be involved in AS development is an open question. Thus, although many proteins are known to be targeted by UBE3A for proteasomal degradation, much less is known about UBE3A interactions with the proteasome itself, or how these interactions might affect substrate turnover, or whether perturbations in this association can contribute to AS development.

The 26S proteasome is a complex cellular machine that contains a 20S central core, a hollow tube composed of multiple proteasome subunits, which contain proteolytic sites. On each end of the 20S proteolytic core, there is an ATP-dependent 19S regulatory particle, which is involved in capturing the ubiquitinated proteins.^28^ Among several subunits that are part of the 19S regulatory particle complex, there are two major ubiquitin receptors, Rpn10/S5a and Rpn13.^29, 30, 31^ The S5a subunit mediates the targeting of ubiquitin substrates to the proteasome by binding ubiquitin conjugates through a ubiquitin-interacting motif (UIM)^32^ and loss of this activity of S5a results in decreased proteolytic activity of the proteasome.^33, 34, 35^ It has also been shown that S5a is regulated by mono-ubiquitination, which inhibits its ability to interact with ubiquitin-conjugated substrates, and also leads to decreased proteasome activity.^31^ Recent studies have shown that UBE3A can directly ubiquitinate the S5a subunit, and that its *Drosophila* ortholog, Ube3a, mediates ubiquitination of the *Drosophila* S5a homolog, resulting in its subsequent degradation.^26, 27^ Structural studies have indicated that a number of AS-associated UBE3A point mutations occur in the HECT domain, which most likely lead to the expression of catalytically defective proteins.^13, 14^ We were therefore interested in investigating whether catalytically defective AS-associated point mutants can still interact with the S5a subunit and, furthermore, in determining whether they can exert any inhibitory effects on the proteasomal turnover of ubiquitinated substrates. We show here that AS-associated UBE3A mutants interact more strongly with S5a, with one of the consequences being a general inhibitory effect on the overall proteolytic activity of the proteasome. These results suggest that perturbation of overall proteasome function may be an important element in the development of AS, which thus shows many similarities with other proteasomal neurogical defects.

## Results

### Angelman syndrome-associated UBE3A mutants retain interaction with the proteasomal S5a subunit

**Figure 1:**
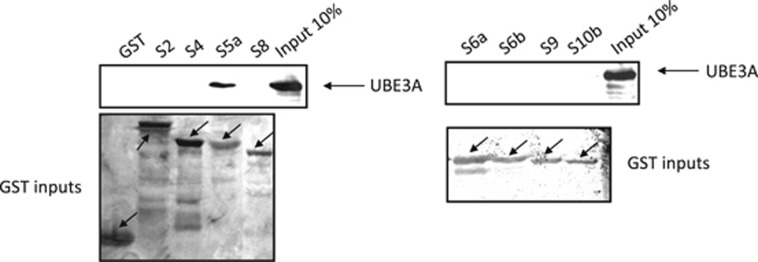
UBE3A interacts with the S5a proteasome subunit. HEK293 cells were transfected with FLAG-tagged wild-type UBE3A expression plasmid and after 24 h cells were collected and extracts incubated with the indicated purified GST fusion proteins. After extensive washing, bound UBE3A was detected by western blotting using the anti-UBE3A antibody and is compared with UBE3A present in 10% of the input. The lower panels show the Ponceau stains of the nitrocellulose membranes showing the levels of GST proteins used in the pull downs with the arrows indicating the position of the GST and GST fusion proteins

Recent studies have shown that UBE3A can directly interact with the S5a proteasomal subunit or can mediate between this subunit and other molecules,^26, 36^ indicating that S5a is a potential proteolytic target of UBE3A.^27^ As mass spectrometric analyses identified multiple 19S regulatory subunits (data not shown)^37^ as potential interacting partners of UBE3A, we were first interested in investigating if any of those other subunits, along with S5a, could also directly interact with UBE3A. To do this, we performed a series of GST pull-down assays using a panel of GST-tagged 19S regulatory subunits. HEK293 cells were transfected with the wild-type FLAG-tagged UBE3A and pull-down assays were performed on the cell extracts 24 h post transfection. Interaction assays were performed using GST fusion proteins of the S2, S4, S5a, S6a, S6b, S8, S9 and S10b subunits, or GST alone for control. The results obtained are shown in Figure 1, where it can be seen that UBE3A only interacts directly with the S5a subunit. This indicates that the capacity of UBE3A to interact with the proteasome is exclusively via the S5a subunit and that the other subunits that were identified in mass spectrometric analyses were most likely detected as a part of the entire proteasomal complex.

### UBE3A catalytically defective mutants interfere with the proteolytic activity of the proteasome

**Figure 2:**
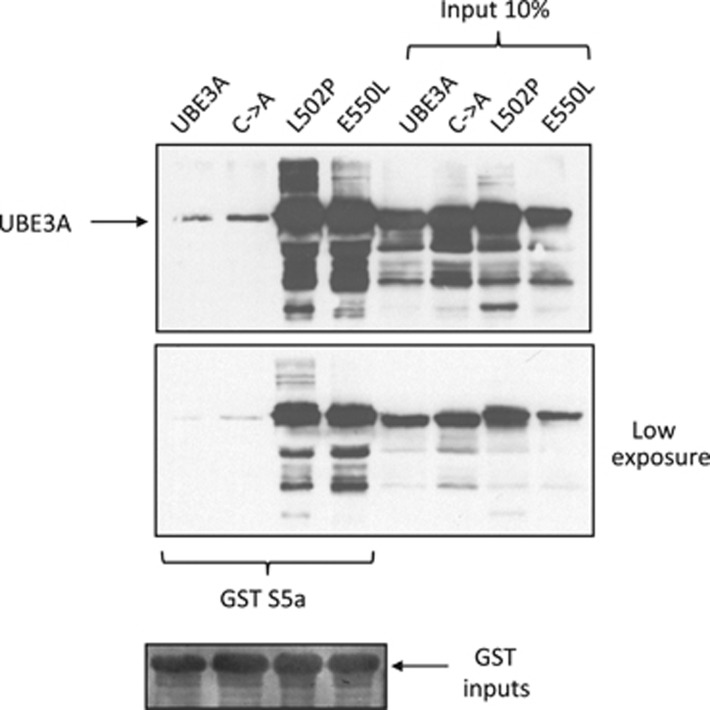
Angelman syndrome mutants strongly interact with the S5a proteasome subunit. HEK293 were transfected with plasmids expressing the wild-type UBE3A and the C833A, L502P, and E550L mutants. After 24 h the cells were harvested and extracts incubated with the S5a GST fusion protein. After extensive washing, bound UBE3A proteins were detected by Western blotting using the anti-UBE3A antibody and are compared with the amount of UBE3A proteins present in 10% of the inputs. The lower panel shows a low exposure of the binding assay and in the bottom panel, Ponceau stain of the nitrocellulose membranes shows the levels of GST proteins used in the pull downs, with the arrows indicating the position of the GST–S5a fusion protein

Since UBE3A is an E3 ubiquitin ligase, it was of interest to determine whether its catalytic activity had a role in the interaction with S5a. To do this we analyzed three catalytically inactive mutants of UBE3A. One mutant, C833A, was artificially generated and is defective in forming a thio-ester bond with ubiquitin *in vitro*^38^ and two are AS-associated point mutants, L502P and E550L, which are defective in ubquitination of HHR23A.^14^ We transfected wild-type and mutant UBE3A expression plasmids into HEK293 cells, and, 24 h post transfection, the cells were extracted and interaction assays performed using the GST–S5a fusion protein. Bound UBE3A was detected by Western blotting and the results in Figure 2 demonstrate that the wild-type UBE3A and the C833A mutant bind S5a to similar degrees, whilst the two AS-associated mutants display a greatly enhanced capacity to interact with the S5a subunit.

**Figure 3:**
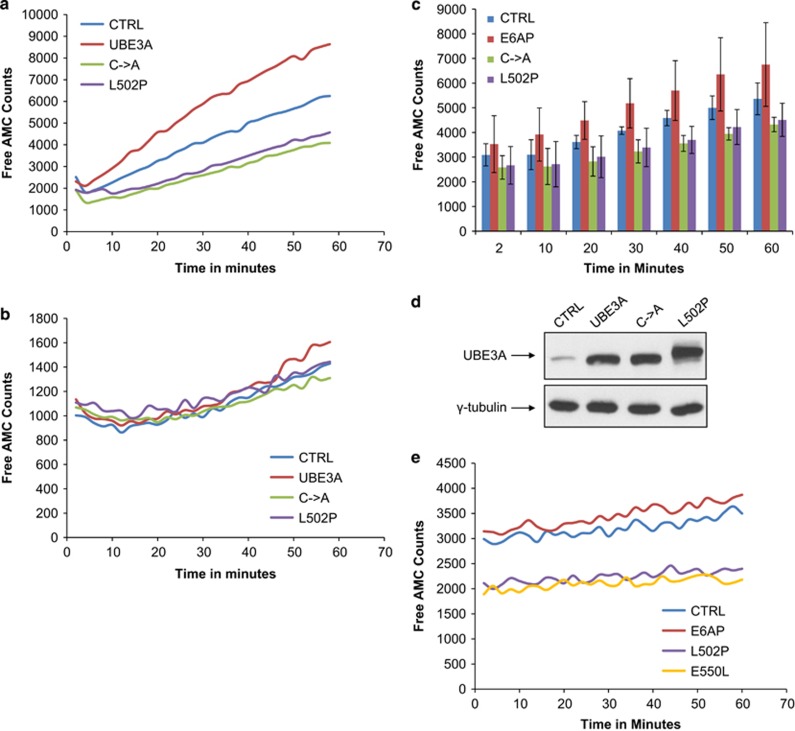
UBE3A wild-type, C->A, L502P and E550L mutants affect the proteolytic activity of the proteasome. HEK293 cells were transfected with UBE3A, C833A, L502P or empty vector as a control (**a**, **b** and **c**) or with UBE3A, L502P, E550L or empty vector as a control (**e**). Twenty-four hours post transfection, cells were collected and homogenized with 0.5% NP40 in dH20, and the proteolytic activity of the proteasome was assessed using the Proteasome Activity Assay Kit and a fluorescent microplate reader in the absence (**a** and **e**) or presence (**b**) of a proteasomal inhibitor. The collated results from three independent experiments to measure UBE3A effects on catalytic activity of the proteasome are shown in **c** at 10-min time point intervals. Standard deviations are also shown. (**d**) Shows the western blot of the wild-type and mutant UBE3A expression levels in HEK293 cells used in the assay shown in **a**. Also shown is the *γ*-tubulin loading control

On the basis of these results, we were next interested in investigating whether the increased interaction between the AS-associated mutants and S5a might have any effects on the overall activity of the proteasome. To do this, HEK293 cells were transfected with UBE3A expression plasmids or the empty vector alone as control. After 24 h, cells were extracted in 0.5% NP40 and homogenized, and the levels of UBE3A expression were verified by western blotting (Figure 3d). The proteasome proteolytic activity was assessed by the Proteasome Activity Assay Kit using a fluorescent microplate reader. The results in Figure 3a show the effects of UBE3A and the two mutants on proteasomal proteolytic activity, while Figure 3b shows their effects upon proteasome activity in the presence of a proteasome inhibitor used as control in monitoring proteasome activity. The combined data from three independent assays, with quantification, are shown in Figure 3c with Free AMC Counts followed over a 60-min period. The data used to calculate the plot in Figure 3c are provided in Supplementary Table 1. These results demonstrate that the AS-associated mutant L502P has a marked inhibitory effect on the overall proteolytic activity of the proteasome. Surprisingly, the C883A mutant also showed a similar inhibitory effect on the proteasome. In contrast, the wild-type UBE3A protein demonstrated an opposite effect from the mutants, and its presence stimulated overall proteasomal proteolytic activity.

We then performed a similar analysis where we assessed the effects of E6AP and the two AS-associated catalytically defective point mutants, L502P and E550L, on proteasome activity. The assays were performed as above, but this time the inputs in the analysis were based on the equal protein concentrations from each transfection, rather than being based on equalizing the levels of E6AP expression. The results in Figure 3e show the effects of wt UBE3A and the AS-associated mutants on proteasomal proteolytic activity where free AMC counts were followed over a 60-min period. The results from three independent experiments are shown in Supplementary Table 2. These results demonstrate, in two different experimental settings, that the AS-associated mutants L502P and E550L have a marked inhibitory effect on the overall proteolytic activity of the proteasome.

### UBE3A affects the proteolytic activity of the proteasome through the S5a subunit

**Figure 4:**
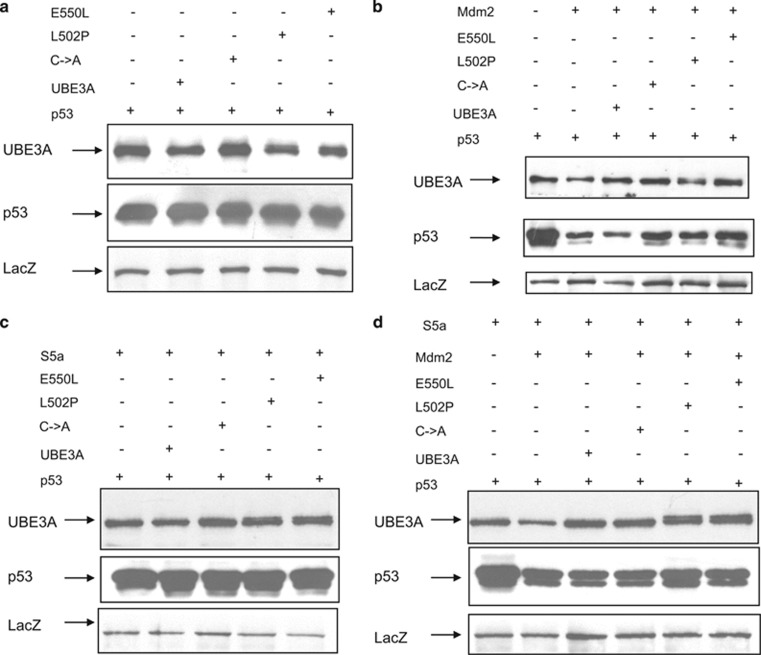
Ectopic expression of S5a rescues UBE3A mutant inhibition of proteasome activity. p53 null H1299 cells were transfected with UBE3A, C833A, L502P, E550L and p53 (**a**, **b**, **c** and **d**), or Mdm2 (**b** and **d**) or S5a (**c** and **d**), alone or in combination. After 24 h, cells were collected, and residual p53 and UBE3A were detected by western blot analysis using either anti-UBE3A antibody or anti-p53 antibody. The expression of *β*-galactosidase (LacZ) was used as a control of transfection efficiency and loading (lower panels)

The above results suggest that UBE3A can exert a direct effect on the overall proteolytic activity of the proteasome, probably via its interaction with the S5a subunit. To investigate this in more detail, we decided to analyze the effects of UBE3A upon specific ubiquitin/substrate combinations: first, we used p53 and Mdm2. To do this, p53 null H1299 cells were transfected with p53 in the presence or absence of Mdm2 and exogenously added UBE3A wild type or mutants, and total cell extracts analyzed by western blotting. The results in Figure 4a demonstrate that neither UBE3A nor the mutants affect the levels of p53 in the absence of exogenous Mdm2. However, when Mdm2 was coexpressed with p53 (Figure 4b), UBE3A wild type slightly increased the ability of Mdm2 to induce the degradation of p53, consistent with the increase in overall proteasome activity, as seen above. Similarly, in the presence of the AS-associated UBE3A mutants, the ability of Mdm2 to degrade p53 was significantly reduced, consistent with their overall negative effect upon proteasome activity. To ascertain whether this was due to inhibition of S5a function, we repeated the experiment in the presence of ectopically expressed S5a. As can be seen (Figure 4c), this reversed the inhibiting effects of the AS-mutants, suggesting that the effects of these mutants on proteasomal activity is via inhibition of S5a.

**Figure 5:**
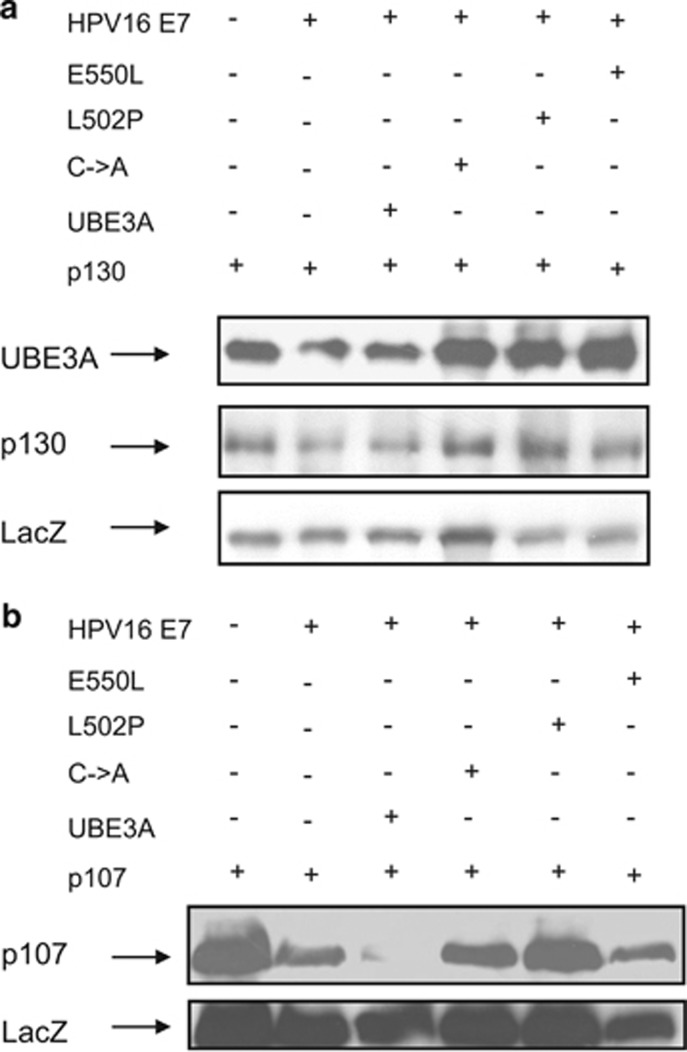
UBE3A catalytically inactive mutants inhibit HPV-16 E7 degradation of p130 and p107. HEK293 cells were transfected with HA-tagged HPV-16 E7, UBE3A, C833A, L502P, E550L (**a** and **b**), or p130 (**a**) or p107 (**b**), alone or in combination. After 24 h, cells were collected, and residual p130, p107 and UBE3A were detected by western blot analysis using either anti-HA antibody, or anti-UBE3A antibody. The expression of *β*-galactosidase (LacZ) was used as a control of transfection efficiency and loading

We then performed a similar analysis on a different set of substrates in a different cell type. It has been previously shown that high-risk HPV-16 E7 oncoprotein mediates proteasome degradation of the pocket proteins p107, p130 and pRb.^39^ We were therefore interested in investigating if the UBE3A mutants have the same inhibitory effect on HPV-16 E7-induced degradation of these proteins. HEK293 cells were transfected with p130 or p107 in the presence or absence of HPV-16 E7, and the wild-type and mutant UBE3A, and total cell extracts analyzed by western blotting. As can be seen in Figure 5, HPV-16 E7 induced the degradation of p130 (Figure 5a) and p107 (Figure 5b) in the presence and absence of wild-type UBE3A. In contrast, when the catalytically inactive UBE3A mutants are present, the levels of p130 and p107 are restored.

**Figure 6:**
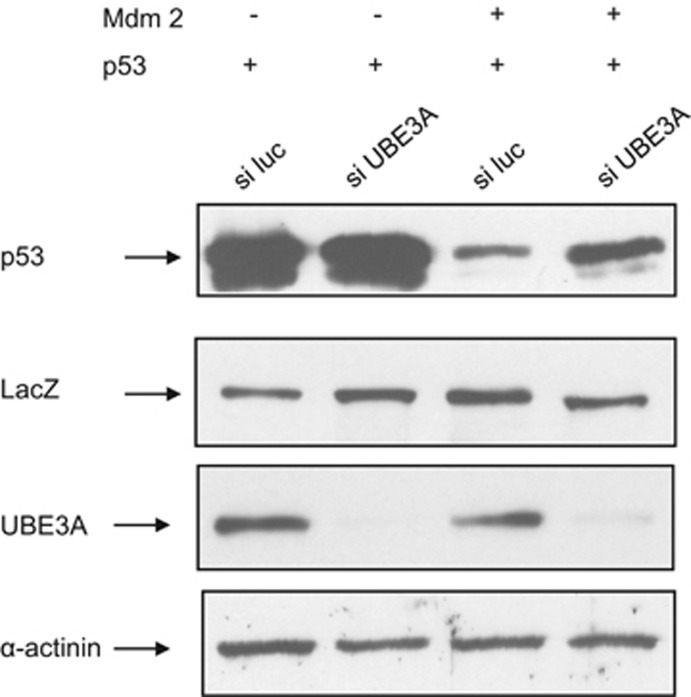
UBE3A knockdown inhibits Mdm2 degradation of p53. p53 null H1299 cells were transfected with siRNA directed against luciferase (si luc) or UBE3A (si UBE3A). After 48 h, cells were transfected with p53 alone or in combination with Mdm2. After 72 h, cells were collected, and the levels of p53, UBE3A and the *α*-actinin and *β*-galactosidase (LacZ) loading controls were detected by western blotting

The above results suggest that UBE3A can affect the overall activity of the proteasome. However, we also wanted to determine whether depleting endogenous UBE3A would affect the ability of Mdm2 to degrade p53. H1299 cells were transfected with siRNA directed against UBE3A or luciferase as a control, and, 48 h later, the cells were further transfected with p53 with or without Mdm2. After a further 24 h, the levels of p53 and UBE3A were then analyzed by western blotting. The results in Figure 6 show that siRNA ablation of UBE3A does not affect p53 levels in the absence of Mdm2, whereas loss of UBE3A in the presence of Mdm2 results in a significant increase in p53 levels. These results are in accordance with the results shown above and demonstrate that UBE3A may be a rate-determining factor in the proteolytic capacity of the proteasome.

### Catalytically inactive UBE3A mutants enhance the S5a subunit ubiquitination

**Figure 7:**
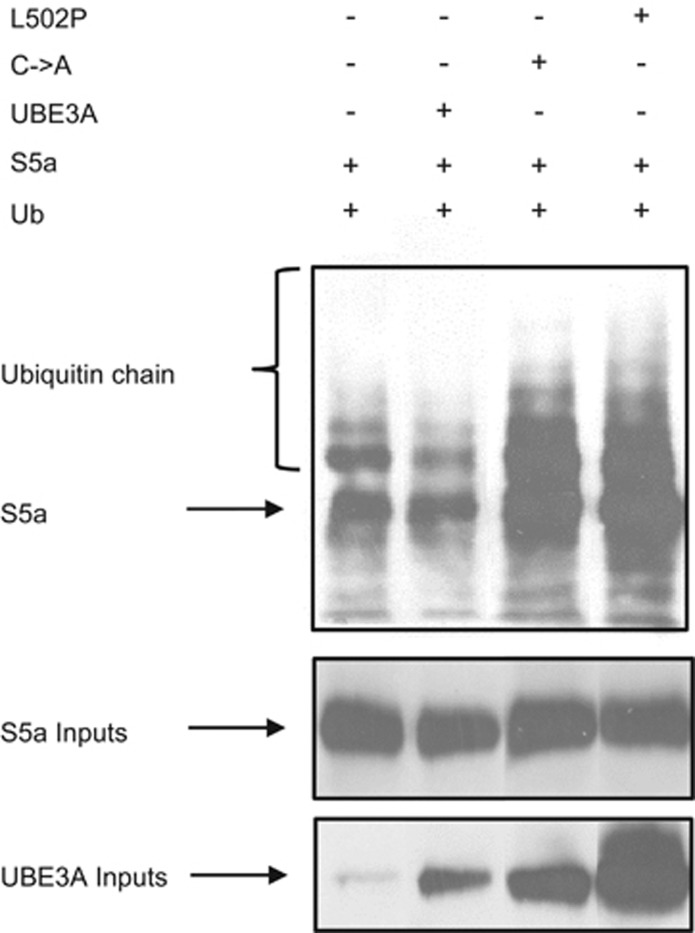
L502P and C833A UBE3A mutants increase S5a ubiquitination. HEK293 cells were transfected with HA-tagged ubiquitin, S5a, UBE3A wild-type, C833A or L502P, alone or in combination. After 24 h, cells were collected and complexes were immunoprecipitated with anti-HA-conjugated agarose beads. Complexes were then analyzed by western blotting for S5a using anti-S5a antibody. Inputs (10%) for S5a and UBE3A are also shown

To identify a potential mechanism by which the catalytically inactive mutants of UBE3A inhibit S5a function, we monitored their effects on the levels of S5a ubiquitination. HEK293 cells were cotransfected with plasmids expressing HA-ubiquitin and S5a, together with plasmids expressing either UBE3A wild-type, C883A, or L502P. Twenty-four hours post transfection, cells were collected in E1A lysis buffer, and complexes were immunoprecipitated using anti-HA-conjugated agarose beads. HA-ubiquitin-bound S5a was then detected by western blotting with anti-S5a antibodies. The results in Figure 7 show clear coimmunoprecipitation of S5a with ubiquitin, which does not increase significantly in the presence of ectopically expressed wild type UBE3A. However, in the presence of the C883A and L502P mutants, the levels of S5a ubiquitination are dramatically increased, suggesting that these catalytically inactive mutants can enhance the accumulation of ubiquitin bound to the S5a, most likely through a perturbation of normal ubiquitin recycling and substrate degradation by the proteasome.

## Discussion

Loss of functional UBE3A expression is associated with the development of AS.^8^ Although most AS cases display a complete loss of UBE3A, there are a number of cases having only point mutations that yet still develop AS pathology.^12, 13^ However, all of these mutations render UBE3A catalytically inactive.^14^ Being an E3 ubiquitin ligase, UBE3A has an important role in the regulation of a number of cellular substrates, and it is reasonable to expect that some of these may be relevant for the development of AS.^15, 21, 22^ However, UBE3A interacts with other components of the proteasome degradatory pathway including other ubiquitin ligases and elements of the proteasome machinery itself, indicating that there may be other means by which UBE3A loss might contribute to the development of AS.^23, 24, 25, 26^ Of particular relevance is the overall functional integrity of the proteasome, loss of which is a common feature in many neurogical disorders. Here, we show that AS-associated catalytically inactive mutants of UBE3A have an increased capacity to interact with the proteasomal subunit S5a. Furthermore, we show that one potential consequence of this increased association is an overall reduction in proteasome activity. These results suggest that loss of functional UBE3A expression, in addition to perturbing the turnover of its known cellular substrates, can also have more far reaching effects on general proteasome function and hence on the normal cellular homeostasis.

A number of studies have shown that UBE3A can interact with the S5a proteasome subunit leading to its polyubiquitination.^26, 27^ Proteomic analyses indicated that UBE3A might associate with other proteasomal components, suggesting a potentially more complex pattern of association. To investigate this, we performed interaction assays between UBE3A and a panel of purified proteasome regulatory subunits. In contrast to the proteomic analyses, we found that UBE3A exclusively interacts with the S5a subunit, and no interaction with the other subunits was observed. This supports previous studies showing a direct interaction between UBE3A and S5a, and suggests that the other potential proteasome component interacting partners of UBE3A identified in proteomic screens are indirect interactions. When we compared wild-type UBE3A with catalytically inactive mutants, a number of interesting features were observed. First, the synthetic catalytic mutant, C833A bound to S5a in a manner similar to the wild-type UBE3A. This alone suggests that the interaction between UBE3A and S5a does not require UBE3A catalytic activity. Most interestingly, the two AS-associated mutants E550L and L502P, which are also both defective in catalytic activity, interacted much more strongly than the wild-type protein with the S5a subunit. One potential explanation for this comes from the crystal structure of the Hect domain of E6AP, which happens to be an L-shaped molecule made of two lobes.^13^ Both of the Angelman-associated mutations, L502P and E550L, are located in the amino-terminal lobe of the HECT domain and their mutations could affect the overall structure of the HECT domain which might lead to defects in HECT domain activities. One consequence of this could be a locking of the HECT domain into a fixed conformation, such that the UBE3A/S5a turnover is blocked, resulting in an apparent increase in association, and thereby blocking access of other substrates to the proteasome.

Indeed, using proteasome activity assays, we showed that ectopically expressed UBE3A upregulates overall proteasomal proteolytic activity, whereas the catalytically inactive mutants of UBE3A have an inhibitory effect on the proteasome. The inhibitory effects on proteasome function were also confirmed using a series of specific ubiquitin ligase/substrate *in vivo* degradation assays. In three cases, p53 degradation by Mdm2, and p130 and p107 degradation induced by HPV-16 E7, all were found to be increased in the presence of wild-type UBE3A. This was further confirmed by siRNA ablation of UBE3A, which also resulted in decrease in the degradation of p53 by Mdm2. In contrast, in each case, the catalytically inactive mutants of UBE3A all had an inhibitory effect on proteasomal activity. Interestingly, this inhibition was reversed by the addition of ectopically expressed S5a, suggesting that the block in the proteasome function was at the level of S5a. As UBE3A appears very closely linked to S5a function, it seems likely that catalytically active UBE3A might be able to promote ubiquitin recycling at the proteasome, and thereby provide a general stimulatory effect on overall proteasome function. Loss of UBE3A or acquisition of mutations inhibiting proteasome activity may occur through different mechanisms. Previous studies have demonstrated that UBE3A increases the degradation of chaperone-bound substrates, and UBE3A expression is increased under various stress conditions, with its overexpression protecting against endoplasmic reticulum stress-induced cell death.^40^ Therefore, it has been suggested that UBE3A also functions as a cellular quality control ubiquitin ligase. On the basis of this, our results suggest that under conditions where expression of UBE3A is lost or where there is simple catalytic inactivation of the UBE3A, such as with the C833A mutation, there could be an accumulation of chaperone-bound substrates, resulting in protein aggregation and inhibition of proteasome function. In contrast, with the AS-associated catalytically inactive mutants that we have analyzed, which have a greatly increased capacity to associate with S5a, this might be expected to directly block the functioning of the S5a subunit. In either case, the net result is an increase in the levels of S5a polyubiquitination. Interestingly, the inhibition of proteasome activity with MG-132 results in a pattern of S5a ubiquitination very similar to that seen with the catalytically inactive UBE3A mutants (data not shown), further supporting the idea that proteasome function can be blocked by decreased ubiquitin turnover on the S5a subunit. Ultimately, this suggests the importance of a functional UBE3A for optimal proteasome function, and therefore implies one way in which lack of either the protein (ablated expression) or its function (catalytically inactive mutation) can contribute not only to AS pathogenesis, but possibly also to the pathogenesis of other neurological diseases which occur due to protein aggregation.

## Materials and methods

### Cells and transfections

HEK293 cells and H1299 cells were grown in Dulbecco's modified Eagle's medium (DMEM) supplemented with 10% fetal bovine serum (FBS). Transfections were done using calcium phosphate precipitations.^41^

### Plasmids

The pCDNA-3 UBE3A (isoform I) and C833A (isoform I), His-tagged pCDNA-4 L502 (isoform III) and E550L (isoform III), pCDNA-3 Mdm2, Flag-tagged pCDNA-3 p53, Flag-tagged and HA-tagged CMV HPV-16 E7 and pCDNA-3 pS5a plasmids have been described previously,^14, 25, 42, 43, 44, 45^ as have the GST fusion proteins (pGEX-4T-2) of S2, S4, S5a, S6a, S6b, S8, S9 and S10b proteasome subunits.^46^ HA-tagged pCDNA-3 p130 and p107 expression plasmids were kindly provided by James DeCaprio.

### Antibodies

The following antibodies were used: anti-Flag mouse monoclonal antibody M2 (Sigma, St. Louis, MO, USA), mouse anti-UBE3A (BD Transduction Labs, San Jose, CA, USA), anti-HA monoclonal antibody 12CA5 (Roche, Basel, Switzerland), mouse anti-p53 DO-1 (Santa Cruz, Dallas, TX, USA), mouse anti-*γ*-tubulin (Sigma), rabbit anti-*α*-actinin (Santa Cruz), anti-*β*-galactosidase (Promega, Madison, WI, USA) and appropriate secondary antibodies conjugated to horseradish peroxidase (HRP; Dako, Glostrup, Denmark).

### GST pull-down assays

Using cellular extracts, pull-down assays were performed by incubating GST fusion proteins immobilized on glutathione agarose beads with cells extracted in E1A buffer (25 mM HEPES pH 7.0, 0.1% NP-40, 150 mM NaCl) plus protease inhibitor cocktail set I (Calbiochem, San Diego, CA, USA) for 1 h at 4 °C on a rotating wheel. After the incubation the beads were washed once briefly, followed by three additional washes with E1A buffer for 10 min on a rotating wheel at 4 °C, as described previously.^47^ After the final wash, the bound proteins were detected using SDS-PAGE and western blotting. The purity and intensity of all fusion proteins was determined by Coomassie Brilliant Blue R (Sigma) staining.

### Western blotting

Total cellular extracts were prepared by directly lysing cells from 6-cm^2^ or 10-cm^2^ dishes in SDS lysis buffer, and protein detections were done as described previously (Tomaić *et al.*^42^).

### siRNA experiments

For transient small interfering RNA (siRNA) experiments, H1299 cells were seeded in 6-cm^2^ dishes and transfected using Lipofectamine RNAiMax (Invitrogen, Carlsbad, CA, USA) with siRNA against luciferase (Dharmacon, Lafayette, CO, USA) as a control and siRNA against E6AP (Dharmacon).

### Proteasome activity assays

Twenty-four hours post transfection, cells were homogenized with 0.5% NP40 in dH_2_0 and cell lysates were processed according to the manufacturer's instructions (Abcam – Proteasome Activity Assay Kit, Cambridge, UK). Equal protein amounts of each lysate were added in duplicates to a pair of wells of an opaque white microwell plate. The volume of each plate was brought to 100 *μ*l with assay buffer. In addition, 10 *μ*l of the positive control was added to paired wells. The volume of these wells was also brought to a total of 100 *μ*l by addition of assay buffer. At the same time, the standard curve was prepared by adding 0, 2, 4, 6, 8 and 10 *μ*l of AMC standard to a series of microplate wells. The volume was adjusted to 100 *μ*l/well with assay buffer to generate a standard range of 0, 20, 40, 60, 80 and 100 pmol per well of AMC. Then 1 *μ*l of the proteasome inhibitor was added to one of the paired wells (samples and positive control only) and 1 *μ*l of assay buffer to the other well, followed by mixing. In the next step, 1 *μ*l of proteasome substrate was added to all the wells except the standard curve wells, followed by mixing. The microwell plate was then protected from light. The samples were then incubated at 37 °C and the proteasome activity was assessed using a fluorescent microplate reader (PerkinElmer – EnVision, 2104 Multilabel Reader, Waltham, MA, USA) measuring the chymotrypsin-like activity of cell lysates releasing free AMC for 60 min at Ex/Em=350/340.

### Ubiquitination assays

HEK293 cells were transfected with the appropriate plasmids. After 24 h, cellular extracts were prepared by lysing 10 cm^2^ dishes in E1A buffer (25 mM HEPES pH 7.0, 0.1% NP40, 150 mM NaCl), followed by immunoprecipitation using anti-HA-conjugated agarose beads for 2 h. The beads were then washed and poly-ubiquitinated proteins detected by SDS-PAGE and western blotting.

## Abbreviations

AMC: 7-amino-4-methylcoumarin
C/EBP*α*: CCAAT/enhancer binding protein *α*
EDD/UBR5: ubiquitin-protein ligase E3 component N-recognin 5
GST: glutathione S-transferase
HECT: homologous to the E6AP carboxyl terminus
HERC2: probable E3 ubiquitin-protein ligase HERC2
HHR23: human homolog of Saccharomyces cerevisiae Rad23
HPV-16 E7: human papillomavirus-16 E7
Mdm2: mouse double minute 2 homolog
MG-132: Z-Leu-Leu-Leu-al
Np40: Tergitol-type NP40
Ring1B: ring finger protein
SDS: sodium dodecyl sulfate
SDS-PAGE: sodium dodecyl sulfate-polyacrylamide gel electrophoresis
UBE3A: ubiquitin-protein ligase E3A
