# Supplementary material for: Angelman syndrome-associated ubiquitin ligase UBE3A/E6AP mutants interfere with the proteolytic activity of the proteasome
Source: Cell Death Dis. 2015 Jan 29;6(1):e1625–. doi: 10.1038/cddis.2014.572 (PMC4669770; doi:10.1038/cddis.2014.572)
Supplement: Supplementary Table Legends [file cddis2014572x3.doc]

Supplementary Table 1. Effects of UBE3A wild type, C->A and L502P on the proteolytic activity of the proteasome. The proteolytic activity of the proteasome was assessed using the Proteasome Activity Assay Kit and a fluorescent microplate reader. The results from three independent experiments to measure UBE3A effects on catalytic activity of the proteasome as free AMC counts are shown at 2 minute time point intervals over a 60 minute period.

Supplementary Table 2. Effects of UBE3A wild type, L502P and E550L on the proteolytic activity of the proteasome. The proteolytic activity of the proteasome was assessed using the Proteasome Activity Assay Kit and a fluorescent microplate reader. The results from three independent experiments to measure UBE3A effects on catalytic activity of the proteasome as free AMC counts are shown at 2 minute time point intervals over a 60 minute period.
